# Varicella zoster virus acute retinal necrosis following eye contusion: case report

**DOI:** 10.1186/1743-422X-2-77

**Published:** 2005-08-31

**Authors:** Petra Svozílková, Eva Říhová, Pavel Diblík, Pavel Kuthan, Zdeněk Kovařík, Bohdana Kalvodová

**Affiliations:** 1 Department of Ophthalmology, 1^st ^Faculty of Medicine, Charles University, Prague, Czech Republic

**Keywords:** acute retinal necrosis, acyclovir, contusion, corticosteroids, varicella zoster virus

## Abstract

**Background:**

Acute retinal necrosis is a sight-threatening disease caused by the group of herpesviruses. The aim of this paper is to report a case of acute retinal necrosis following ocular trauma in a patient initially treated with vaso-active drugs and corticosteroids for presumed ocular ischemic syndrome.

**Case presentation:**

A 51-years-old otherwise healthy man, who suffered from sudden visual loss in the left eye following contusion, was commenced on vaso-active drugs and systemic corticosteroids for suspected ocular ischemic syndrome with extensive swelling of the optic disc and macular edema. Subsequently, vision in the initially uninvolved right eye decreased. Polymerase chain reaction of vitreous samples and retinal biopsy confirmed varicella zoster virus. Despite intensive treatment with intravenous antiviral medication, the patient became completely blind in both eyes.

**Conclusion:**

Initial treatment of acute, unexplained visual decrease with systemic corticosteroids may lead to visual loss in patients with developing acute retinal necrosis. Ocular trauma could have induced and corticosteroid treatment promoted reactivation of a latent viral infection in our patient.

## 

Acute retinal necrosis (ARN) is a sight-threatening clinical syndrome caused by the group of herpesviruses (herpes simplex virus; HSV-1 and HSV-2, varicella zoster virus; VZV, cytomegalovirus; CMV or Epstein-Barr virus; EBV). Rapidly progressing retinal inflammation leads to severe impairment of vision.

## Case presentation

**Figure 1:**
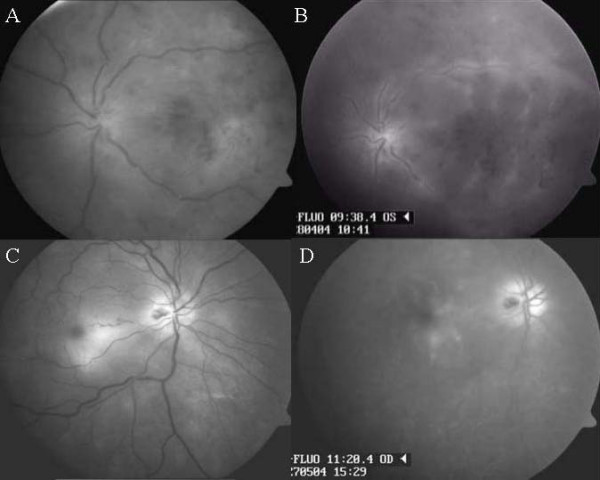
Red free fundus photographs and late phases of fluorescein angiography. (A, B) On initial examination, red free photograph and late phase of fluorescein angiography of the left eye demonstrated swelling of the optic disc, ischemic macular edema, narrowing of the arterioles, dilatation of the venules and superficial retinal hemorrhages. (C, D) Four weeks later, fundus examination of the initially uninvolved right eye revealed swelling of the optic disc with hemorrhages and ischemic macular edema.

We present a case of a 51-year-old otherwise healthy man, who suffered from rapid visual loss in the left eye following contusion. Ocular trauma was caused during a football match by a ball, which hit an index finger located just in front of the bulbus. The patient attended our department on April 27, 2004, one week after the injury, when the vision in the left eye decreased to light perception with inaccurate light projection and hand movements in a lower part of visual field. The best-corrected visual acuity in the right eye was 1.0. Intraocular pressures were 18 mmHg in the right eye and 45 mmHg in the left eye. Examination of the anterior segment and fundus of the right eye revealed no pathology. The left eye showed discrete injection of the conjunctiva and keratic precipitates with mild anterior chamber flare and cells. There was iridodonesis, cleft syndrome and a relative afferent pupillary defect in the left eye. The fundus examination of the left eye revealed swelling of the optic disc, large ischemic macular edema, superficial retinal hemorrhages, narrowing of the arterioles and dilatation of the venules (Figure 1A). Fluorescein angiography of the left eye showed macular edema and vascular leakage in the venous phase (Figure 1B). Duplex Doppler ultrasonography and computed tomography scans of the brain and orbits were normal.

Based on the clinical findings, the presumed diagnosis of ocular ischemic syndrome was made. The patient was initially treated with vaso-active drugs in addition to corticosteroids. Intravenous methylprednisolone (500 mg daily for 5 days) followed by 60 mg of oral prednisone daily was indicated due to swelling of the optic disc and macular edema. Despite intensive therapy, the fundus examination showed progression of ischemic lesions. Visual acuity in the left eye was light perception with inaccurate light projection. The finding on the right eye was without changes. The patient was discharged on oral prednisone 50 mg daily.

On May 24, 2004, four weeks after pulse intravenous corticosteroid treatment, vision in the initially uninvolved right eye decreased to 0.25. Fundus examination of the right eye disclosed swelling of the optic disc with hemorrhages, blurring of the optic disk margins and ischemic macular edema (Figure 1C, D). In the left eye, massive vitreous opacities made evaluation of the fundus impossible.

A differential diagnosis of antiphospholipide syndrome, masquerading syndrome, viral retinitis or specific inflammation was considered.

No neurological or other abnormalities were found on systemic examination. The findings from magnetic resonance imaging and magnetic resonance angiography of brain and orbits were within normal limits. The cerebrospinal fluid was negative for VZV DNA and enteroviruses RNA. Chest X ray and abdominal ultrasonography were normal. Leukocyte count, hematocrit and activated partial tromboplastin time (APTT) were normal, liver tests showed elevated levels of alaninaminotranspherase (ALT; 2.63 ukat/l). Anti-cardiolipin antibodies were negative. Serologic tests for syphilis and human immunodeficiency virus (HIV-1/-2) were negative. Serum was evaluated regarding evidence for herpesviruses by means of polymerase chain reaction (PCR). Low levels of VZV and EBV EBNA-1 IgG antibodies were detected in serum, whereas IgM antibodies were absent; as well antibodies of respiratory infections or neuroinfections were negative. Blood cultures were also negative. Immunofenotypization showed lower count of lymphocytes in peripheral blood, without plasma cell neoplasia.

**Figure 2:**
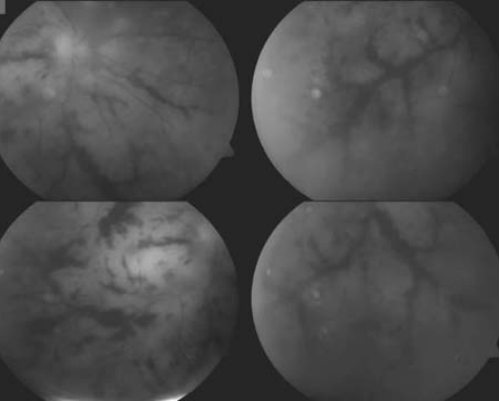
Red free fundus photograph of the right eye after intravenous corticosteroid treatment. Fundus examination disclosed several enlarging foci of necrotizing retinitis.

On May 27, 2004, an aqueous tap of the left eye was performed and samples were submitted for cytological and virological analysis. PCR of aqueous humour was negative for herpesviruses family and cytology confirmed non-purulent intraocular inflammation. The patient was treated with corticosteroids. The best-corrected visual acuity in the right eye decreased to 0.02. Due to progressive impairment of the clinical status, the corticosteroid therapy was stopped. Fundus examination demonstrated several enlarging foci of necrotizing retinitis with extensive posterior pole involvement (Figure 2). Based on the clinical appearance, a diagnosis of presumed necrotizing herpetic retinopathy was made. The patient was commenced on high-dose intravenous acyclovir (4 × 500 mg per day for 2 weeks). Two days later, visual loss with acuity reduced to light perception with inaccurate light projection occurred in the right eye. In the left eye, there was progression of vitreous opacities and the vision was light perception with inaccurate light projection.

On June 4, 2004, a diagnostic pars plana vitrectomy and retinal biopsy were carried out in the left eye. The vitreous cavity was filled with 16% perfluoropropane (C_3_F_8_) gas. A part of retina and samples of diluted and undiluted vitreous were obtained. Due to failure of the antiviral treatment and ocular disease progression, the patient underwent a pars plana vitrectomy in the right eye on June 9, 2004.

PCR of retina of the left eye and undiluted vitreous of both eyes were positive for VZV. Undiluted vitreous was negative for HSV-1 and -2, CMV, EBV. PCR of diluted vitreous was negative for herpesviruses family. Mycobacterium tuberculosis was not detected using PCR in vitreous of both eyes. Cultivation of vitreous for bacteria and fungi was negative; *Toxoplasma gondii *antibodies were also negative. Histopathological examination confirmed non-purulent intraocular inflammation. Immunofenotypization of vitreous of both eyes showed no plasma cell neoplasia.

In two weeks, intravenous acyclovir was followed by oral acyclovir (5 × 400 mg daily). In the right eye, large foci of retinal atrophy with reduced inflammatory reaction were present. Owing the cataract induced by gas, fundus of the left eye was not visible.

The patient was discharged on acyclovir 4 × 400 mg daily. However, an exudative retinal detachment was seen in the right eye and vision decreased to 0. Vision in the left eye was light perception with inaccurate light projection. On examination 4 weeks later, B-scan ultrasonography of the left eye confirmed the exudative retinal detachment. Nevertheless, despite intensive treatment with intravenous antiviral medication, the patient became completely blind in both eyes.

## Discussion

ARN is a serious ophthalmic manifestation of infection caused by the herpesviruses family. A rapid and accurate diagnosis of herpetic infection is crucial for prompt administration of specific antiviral therapy. Although the precise pathogenesis of ARN is not completely understood, Kezuka and coworkers [1] found out that a high proportion of patient with ARN associated with VZV displayed a transient loss of virus-specific delayed hypersensitivity, but their serum samples contained high titers of anti-VZV antibodies. The authors propose that idiopathic reactivation of VZV in one eye might promote suppression of delayed hypersensitivity, thereby eliminating the virus-specific CD4+ T cells that are required to prevent neural spread of the virus from the site of reactivation. ARN in the contralateral eye may be the inevitable consequence. On resolution of the intraocular inflammation, virus-specific delayed hypersensitivity recurred in most of these individuals. Patients with ARN syndrome should be followed because of possible recurrence.

We present a case of a 51-year-old otherwise healthy man with rapid visual loss initially treated with vaso-active drugs and systemic corticosteroids for presumed ocular ischemic syndrome with swelling of the optic disc and macular edema. The causative agent was diagnosed as VZV based on PCR analysis of vitreous and retinal samples. Possible mechanisms of VZV necrotizing retinopathy include reinfection by an exogenous virus or reactivation of a latent infection. In our opinion, ocular trauma probably induced reactivation of a latent virus in the presented patient. Absence of high VZV titers in the serum makes systemic reinfection unlikely. Thompson and coworkers [2] demonstrated three patients treated for ARN apparently caused by reactivation of latent HSV-2. Primary viral infection was probably congenital, with documented perinatal HSV-2 infection in two patients. In all these cases, periocular trauma preceded the development of retinitis by two to three weeks. To our knowledge, the possible reactivation of VZV by ocular trauma has never been reported.

A unique case of acute HSV encephalitis associated with bilateral ARN syndrome after craniotomy for resection of a suprasellar craniopharyngioma has been reported. The authors hypothesized reactivation of previously latent HSV in the area of the inferior frontal lobe and optic chiasm. Reactivated virus may have migrated to the retina by axonal transport, through the optic nerves, to induce the ARN syndrome [3]. The onset of bilateral necrotizing herpetic retinopathy three years after HSV encephalitis following pulse corticosteroid treatment has also been described. Based on the extremely rapid development of retinitis to involve the fellow eye after pulse corticosteroid therapy, the authors concluded that treatment with corticosteroids alone might increase the risk of reactivation of latent infection [4].

Ocular trauma could have induced, and systemic corticosteroid treatment probably promoted, reactivation of a latent virus in our patient. Initial treatment of acute, unexplained decrease of vision with systemic corticosteroids may lead to visual loss in patients with developing necrotizing herpetic retinopathy [5]. Since progression to profound and irreversible visual loss is rapid, early diagnostic vitreous biopsy must be performed before commencement of immunosuppressive drugs. PCR analysis of vitreous samples is a valuable tool in the early diagnosis and initiation of appropriate treatment.

## Competing interests

The author(s) declare that they have no competing interests.
